# Phenobarbital use in benzodiazepine and z-drug detoxification: a single-centre 15-year observational retrospective study in clinical practice

**DOI:** 10.1007/s11739-022-02976-0

**Published:** 2022-04-12

**Authors:** Simone Sartori, Giada Crescioli, Valentina Brilli, Sara Traversoni, Cecilia Lanzi, Alfredo Vannacci, Guido Mannaioni, Niccolò Lombardi

**Affiliations:** 1 Department of Neurosciences, Psychology, Drug Research and Child Health, Section of Pharmacology and Toxicology, University of Florence, Viale G. Pieraccini, 6, 50139 Florence, Italy; 2 Medical Toxicology Unit and Poison Control Centre, Careggi University Hospital, Florence, Italy; 3 Tuscan Regional Centre of Pharmacovigilance, Florence, Italy

**Keywords:** Phenobarbital, Benzodiazepine, Z-drug, Clinical practice, Detoxification, Toxicology

## Abstract

Given the increase in benzodiazepine (BZD) and Z-drug (ZD) use disorder, this study described the use of phenobarbital (PHB) as detoxification in clinical practice. A 15-year observational retrospective study was performed on medical records of BZD-ZD use disorder patients detoxified with PHB at the Toxicology Unit and Poison Centre, Careggi University Hospital, Florence (Italy). A multivariate logistic regression was used to estimate odd ratios (ORs) and related 95% confidence intervals (CI) of “treatment failure” considering demographic and pharmacological characteristics. “Hospitalisation length”, “PHB discharge dose”, and “BZD-ZD free status” at discharge were also calculated. During detoxification, out of 355 patients (57% of men), with a mean age of 42.92 years, only 20 (5.6%) treatment failures were recorded: 19 were discharged against medical advice or due to misbehaviour, and only one for PHB-related non-serious skin rash. Analysis showed a higher probability to be BZD-ZD free at discharge for subjects who reported to be employed (OR 2.29; CI 95% 1.00–5.24), for those who abused oral drops of BZD-ZD (OR 2.16, CI 1.30–3.59), and for those treated with trazodone (OR 2.86, CI 1.14–7.17) during hospital stay. A hospitalisation length of > 7 days was observed for patients with opioid maintenance therapy (OR 2.07, CI 1.20–3.58) for substance use disorder, and for those treated with more than 300 mg/day of PHB equivalents at hospital admission (OR 1.68, CI 1.03–2.72). Our results suggested that PHB can be considered a valuable detoxification option for different types of BZD and ZD use disorder patients.

**Supplementary Information:**

The online version contains supplementary material available at 10.1007/s11739-022-02976-0.

## Introduction

Benzodiazepines (BZD) are a group of drugs with anxiolytic, hypnotic, sedative, anti-seizures, and muscle-relaxant properties, first discovered in 1957 when chlordiazepoxide was synthesised [1]. This class of medications modulates several central nervous system (CNS) inhibitory pathways, mainly as positive allosteric modulators for γ-aminobutyric acid (GABA)-A receptor, facilitating GABA binding to this chloride channel.

BZD have a better safety profile for CNS depression when compared to barbiturates [1], and this brought to their wide acceptance in clinical use for brief symptomatic treatments of anxiety, insomnia, and panic disorders, until chronic and more stabilised therapies reach their steady levels. Other recognised medical uses for BZD are emergency treatment of seizures and alcohol use disorder treatment. Nevertheless, BZD can cause tolerance after 4 up to 6 weeks of use [2], resulting in possible physical dependence, that usually appears in case of abrupt cessation, with life-threatening conditions such as seizures and delirium. BZD also carry significant complications, such as memory impairment, vehicle accidents, falls, and overdose, and they are among the most frequent prescription drugs prone to misuse, abuse and diversion (along with opioids) [3].

It was only at the end of the 80’s that a new class of drugs was discovered, similar to BZD as for the clinical effects but completely different at a molecular level. They were named as non-benzodiazepines, also known as z-drugs (ZD), due to the names given to the first compounds discovered (e.g., zolpidem, zopiclone, zaleplon).

Overall, the need for safe and effective detoxification protocols from sedative-hypnotic medications has increased as the availability both via prescription and illicit markets has increased dramatically in recent years [3]. The COVID-19 pandemic only compounded this as rates of BZD prescriptions increased during this period and continue to remain elevated [4]. For the above reasons, discontinuing sedative-hypnotic medications should be considered after prolonged use, especially if used improperly or abused [5].

The current guidelines for BZD and ZD use disorder treatment recommend a long-acting BZD tapering [2, 6, 7], although this method requires long periods of outpatient monitoring and it still carries the chance of precipitating withdrawal symptoms in the final steps of detoxification [6]. Other methods have been proposed to discontinue BZD and ZD, ranging from pharmacological management with varying degrees of efficacy (e.g., antagonism with low dose flumazenil, cross-tolerance tapering with phenobarbital, non-BZD withdrawal control with carbamazepine or topiramate) to a psychological approach with cognitive behaviour therapy [1]. Overall, there is still no treatment consensus as far as BZD and ZD use disorder is concerned and, despite the social and health importance of this issue, no substantial pharmacological progresses have been made in this toxicological field. Furthermore, choosing a different detoxification protocol often involves consideration of substance type, comorbidities, co-abuses, and the need for a specific clinical setting (inpatient and/or outpatient).

As BZD and alcohol, phenobarbital (PHB) binds GABA-A receptors and has been used for the treatment of alcohol withdrawal. However, evidence on its efficacy and safety in the treatment of BZD and ZD disorders is scarce, and its use in this clinical context in past years was limited, mainly for potential adverse drug reactions (ADRs) [1, 6]. In this context, the aim of the present study was to describe the use of PHB, alone or in combination with long half-life benzodiazepines (BZD), in BZD and z-drugs (ZD) use disorder patients. Demographic and clinical characteristics of patients, as well as the effectiveness and tolerability of PHB were also described.

## Methods

**Fig. 1:**
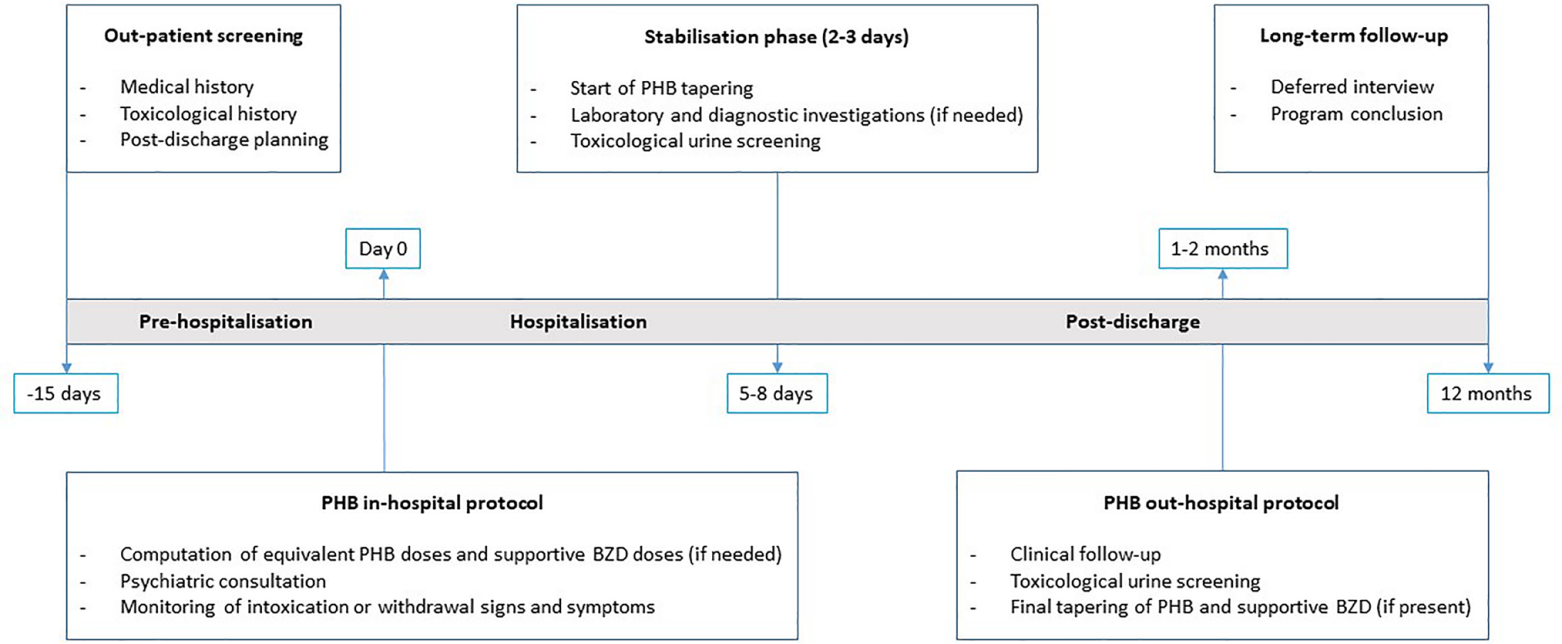
Phenobarbital-based detoxification treatment

An observational retrospective study was carried out on BZD and/or ZD use disorder patients admitted at the Medical Toxicology Unit and Poison Control Centre of Careggi University Hospital (Florence, Italy), analysing their electronic medical records from January 1st, 2006 to December 31st, 2020. All BZD and/or ZD use disorder patients referring to the Medical Toxicology Unit underwent the same detoxification treatment based on PHB (maximum dose of 300 mg/day). This is the standard treatment performed at the Medical Toxicology Unit in the last decades, and its characteristics are depicted in Fig. 1. In addition to the use of PHB alone, the detoxification treatment included supportive BZD with long half-life, such as chlordiazepoxide (CZD), diazepam or delorazepam, especially when further withdrawal covering was required.

All demographic and clinical data were extracted from medical charts by two trained clinical toxicologists, anonymized and collected in an ad hoc database. A detailed medical and toxicological history was collected, involving educational, occupational and pregnancy status, comorbidities, concomitant medications, and specifics about patients’ BZD and/or ZD use disorder. In particular, the following items were analysed: active substance abused and its average daily dosage (mg/day); pharmacokinetics features (formulation and plasma half-life); misuse/abuse duration; administration route; psychiatric comorbidities (mood, anxiety, sleep, psychotic, or personality disorders), if present; co-abuse (alcohol, opioid, cocaine or their association), and its treatment if present. Patients with concomitant co-abuse were considered as “polyabusers”. Smoking was not considered within co-abused substances. Finally, completion of the detoxification treatment as an outpatient through ambulatory visits during the SARS-CoV-2 pandemic was also recorded. Due to the retrospective nature of the present study and data anonymization, patient’s consent to participate was not required.

To compare abuse severity, each BZD and ZD was paired with its defined daily dose (DDD, when available) [8], and its referred total daily intake. Average dose was measured converting the BZD and ZD total daily intake into diazepam equivalent dose, according to tables available in literature. The equivalent dose of PHB was recorded from patients’ abuse history, according to tables available in literature [9–11]. When more than one active substance among BZD and ZD was concomitantly taken, the one with the highest daily intake was considered for computing substance plasma half-life preference in our population. No specific differences were made between methadone or buprenorphine in concomitant opioid use disorder, and they were included altogether under the term “opioid maintenance therapy”.

Symptoms of intoxication (e.g., slurred speech, nystagmus, or ataxia), withdrawal (e.g., tremors, sweating, agitation), and PHB-related ADRs (e.g., systemic dermatological or neurological events) were retrieved from medical records. Concomitant supportive BZD with long half-life, such as CZD, diazepam or delorazepam, and gabapentin (GBP) or trazodone (TZD) used for the treatment of dysphoria or insomnia, were also considered.

The PHB treatment failure was defined as the patient’s dropout from the detoxification treatment (e.g., in case of PHB-related ADR, patient inappropriate behaviour, patient who refuse to continue detoxification). The probability of treatment failure was calculated considering both demographic (gender, age, employment, psychiatric comorbidities, alcohol or drug co-abuse, and opioid maintenance therapy) and pharmacological (PHB equivalents at admission, number of abused substances, plasma half-life, formulation, administration route, and supportive therapy) characteristics. We also calculated the “hospitalisation length” (> 7 days), “PHB discharge dose” (> 100 mg/day), and “BZD free status” at discharge, considering the same demographic and pharmacological characteristics. Specifically, “BZD free status” was defined as the patient’s discharge without the original abused BZD and/or ZD and any supportive BZD administered during the hospitalisation phase.

Descriptive statistics were used to summarise data. Categorical data were reported as frequencies and percentages and compared using the chi-squared test, whereas continuous data were reported as median values with the related interquartile ranges (IQR), or mean and standard deviation (SD), and compared using the Mann–Whitney test or two-tailed *t* Student test, respectively.

Univariate logistic regression was used to estimate the odds ratios (ORs) of “treatment failure” with 95% confidence intervals (CIs). Multivariate logistic regression was performed and adjusted for: age, sex, PHB equivalents, number of abused active substances, plasma half-life and formulation. Multivariate logistic regression models were also used to estimate the ORs and 95% CIs of “hospitalisation length” (> 7 days), “PHB discharge dose” (> 100 mg/day), and “BZD free status” at discharge. All results were statistically significant at *p* value < 0.05. Data management and statistical analysis were carried out using STATA 17.

## Results

The characteristics of the 355 use disorder patients admitted to the Medical Toxicology Unit for BZD and or ZD detoxification were reported in Table 1. The mean age was 42.92 (SD ± 10.66) years, and 57% were men. Two-hundred-seventy-one (76.34%) patients had psychiatric comorbidity, of which 83 (23.38%) presented mood disorders, 93 (26.20%) anxiety, and 67 (18.87%) personality disorders. One-hundred-twenty (33.8%) patients had alcohol co-abuse history, while 103 (29.01%) had other drugs co-abuse. One-hundred-seven (30.14%) patients were under opioid maintenance therapy with buprenorphine or methadone. Of notice, among females, four pregnant women underwent the detoxification treatment. No malformation or other pregnancy/neonatal negative outcomes were reported. Moreover, six patients completed the detoxification treatment as outpatients through ambulatory visits due to COVID-19-related emergency measures.

**Table 1:** Baseline characteristics of benzodiazepine and z-drug use disorder patients

	Total, *N* = 355 (%)	Men, *N* = 203 (%)	Women, *N* = 152 (%)	*P* value
Mean age (± SD)	42.92 ± 10.66	41.92 ± 10.81	44.26 ± 10.34	0.040
Median age (IQR)	42 (36–49)	40 (34–48)	43 (37–51)	0.009
Educational level				
Elementary school	12 (3.38)	8 (3.94)	4 (2.63)	0.253
Middle school	74 (20.85)	49 (24.14)	25 (16.45)	
High school	53 (14.93)	25 (12.32)	28 (18.42)	
University	34 (9.58)	18 (8.87)	16 (10.53)	
Not reported	182 (51.27)	103 (50.74)	79 (51.97)	
Employment				
Employed	56 (15.77)	24 (11.82)	32 (21.05)	
Unemployed/retired	78 (21.97)	42 (20.69)	36 (23.68)	0.028
Not reported	221 (62.25)	137 (67.49)	84 (55.26)	
Psychiatric comorbidity				
Yes	271 (76.34)	136 (67.00)	135 (88.82)	< 0.001
No	84 (23.66)	67 (33.00)	17 (11.18)	
Psychiatric disorder type				
Mood	83 (23.38)	40 (19.70)	43 (28.29)	< 0.001
Anxiety	93 (26.20)	46 (22.66)	47 (30.92)	
Sleep	15 (4.23)	7 (3.45)	8 (5.26)	
Personality	67 (18.87)	33 (16.26)	34 (22.37)	
Psychotic	5 (1.41)	2 (1.32)	3 (1.48)	
Others	8 (2.25)	7 (3.45)	1 (0.66)	
Not reported	84 (23.66)	67 (33.00)	17 (11.18)	
Pregnancy				
Yes	4 (1.13)	–	4 (2.63)	0.020
No	351 (98.87)	–	148 (97.37)	
Alcohol co-abuse				
Yes	120 (33.80)	77 (37.93)	43 (28.29)	0.057
No	235 (66.20)	126 (62.07)	109 (71.71)	
Drug co-abuse				
Yes	103 (29.01)	76 (37.44)	27 (17.76)	< 0.001
No	252 (70.99)	127 (62.56)	125 (82.24)	
Drug type				
Heroin	44 (12.39)	19 (9.36)	25 (16.45)	0.001
Cocaine	27 (7.61)	22 (10.84)	5 (3.29)	
Cannabis	17 (4.79)	10 (4.93)	7 (4.61)	
Association	40 (11.27	31 (15.27)	9 (5.92)	
Other co-abuse				
Yes	19 (5.35)	8 (3.94)	11 (7.24)	0.172
No	336 (94.65)	195 (96.06)	141 (92.76)	
Opioid maintenance therapy				
Yes	107 (30.14)	83 (40.89)	24 (15.79)	< 0.001
No	248 (69.86)	120 (59.11)	128 (84.21)	

Opioid maintenance therapy: buprenorphine or methadone

*IQR* interquartile range, *SD *standard deviation

Equivalent PHB mean converted dose at admission was 1205.76 mg/day (SD ± 1456.57), with an actual given PHB starting dose of 229.31 ± 67.78 for men and of 211.84 ± 66.79 for women (*p* = 0.016), respectively (Table 2). Hospitalisation length was 8.25 (SD ± 3.66) days, with a difference observed between men and women in terms of PHB therapy duration throughout hospital stay (3.74 ± 2.71 vs 4.63 ± 3.37 days; *p* = 0.006). Total PHB duration mean, also considering outpatient management, was 18.57 ± 18.56 days, with a statistically significant difference between men and women (16.84 ± 16.59 vs 20.87 ± 20.73; *p* = 0.043). One-hundred-sixty-four (46.20%) use disorder patients did not require any supportive BZD therapy during detoxification, whereas 150 (42.25%) patients required one long half-life BZD, and 41 (11.55%) required an association of two or more long half-life BZD. At discharge, 230 patients (64.79%) were BZD free, and the others (35.21%) had only long half-life supportive BZD therapy which was monitored and tapered during the post-hospitalisation phase.

**Table 2:** Phenobarbital detoxification and supportive therapy

	Total, *N* = 355 (%)	Men, *N* = 203 (%)	Women, *N* = 152 (%)	*P* value
PHB equivalents at admission (mg/day), mean (± SD)	1205.76 ± 1456.57	1306.55 ± 1647.54	1071.16 ± 1144.89	0.132
Hospitalisation length (days), mean (± SD)	8.25 ± 3.66	8.04 ± 3.39	8.53 ± 3.98	0.213
PHB use, mean (± SD)				
Start dose (mg/day)	221.83 ± 67.82	229.31 ± 67.78	211.84 ± 66.79	0.016
In-hospital treatment (days)	4.12 ± 3.04	3.74 ± 2.71	4.63 ± 3.37	0.006
Discharge dose (mg/day)	90.21 ± 64.31	88.67 ± 64.83	92.27 ± 63.77	0.602
Out-hospital treatment (days)	14.45 ± 17.40	13.10 ± 15.91	16.24 ± 19.13	0.093
Total treatment duration (days)	18.57 ± 18.56	16.84 ± 16.59	20.87 ± 20.73	0.043
Supportive BZD				
Yes	150 (42.25)	85 (41.87)	65 (42.76)	0.160
Association	41 (11.55)	29 (14.29)	12 (7.89)	
No	164 (46.20)	89 (43.84)	75 (49.34)	
Supportive CDZ				
Yes	141 (39.72)	85 (41.87)	56 (36.84)	0.338
No	214 (60.28)	118 (58.13)	96 (63.16)	
Start dose (mg/day), mean (± SD)	52.98 ± 26.53	58.94 ± 26.41	43.93 ± 24.25	0.001
Discharge dose (mg/day), mean (± SD)	29.93 ± 21.40	29.65 ± 21.01	30.36 ± 22.15	0.848
In-hospital duration (days), mean (± SD)	4.12 ± 2.88	3.75 ± 2.64	4.68 ± 3.14	0.061
BZD-ZD free status at discharge				
Yes	230 (64.79)	130 (64.04)	100 (65.79)	0.733
No	125 (35.21)	73 (35.96)	52 (34.21)	
Supportive GBP				
Yes	146 (41.13)	88 (43.35)	58 (38.16)	0.325
No	209 (58.87)	115 (56.65)	94 (61.84)	
Start dose (mg/day), mean (± SD)	363.51 ± 234.20	367.41 ± 254.85	357.62 ± 201.03	0.804
Discharge dose (mg/day), mean (± SD)	803.35 ± 279.09	787.78 ± 285.54	827.12 ± 269.63	0.402
Supportive TZD				
Yes	68 (19.15)	27 (13.30)	41 (26.97)	0.001
No	287 (80.85)	176 (86.70)	11 (73.03)	
Supportive psychiatric therapy				
Anti-depressive	55 (15.49)	24 (11.82)	31 (20.39)	< 0.001
Anti-psychotic	39 (10.99)	23 (11.33)	16 (10.53)	
Stabiliser	28 (7.89)	17 (8.37)	11 (7.24)	
Association	158 (44.51)	80 (39.41)	78 (51.32)	
No therapy	75 (21.13)	59 (29.06)	16 (10.53)	

*BZD* benzodiazepine, *CDZ* chlordiazepoxide, *GBP* gabapentin, *IQR* interquartile range, *PHB* phenobarbital, *SD* standard deviation, *TZD* trazodone, *ZD* z-drug

Phenobarbital treatment failure according to patients’ demographic and pharmacological characteristics is reported in Table 3. Overall, only 20 (5.6%) failures were identified, of whom 19 were referred to discharge against medical advice or to misbehaviour, and only one patient discontinued PHB treatment due to a non-serious ADR (skin rash). Fourteen of the above subgroup resulted suffering from psychiatric comorbidities. Although at a non-statistically significant level, multivariate logistic regression showed that “treatment failure” was higher for the presence of psychiatric comorbidities (OR 1.07, CI 0.36–3.17), opioid maintenance therapy (OR 1.53, CI 0.54–4.30), drops (OR 1.89, CI 0.60–5.97) and oral (OR 1.59, CI 0.18–13.76) administration route, and for the presence of long half-life BZD (OR 1.78, CI 0.67–4.76), GBP (OR 1.49, CI 0.58–3.82) or TZD (OR 1.24, CI 0.39–3.99) as supportive therapies.

**Table 3:** Phenobarbital treatment failure considering demographic and pharmacological characteristics

	Treatment failure		Univariate OR (95% CI)		Multivariate OR (95% CI)
	Yes	No			
Sex					
Men	15	188	1		1
Women	5	147	0.43 (0.15–1.20)		0.47 (0.15–1.42)
Age					
> 40 years	12	130	1		1
≤ 40 years	8	205	0.42 (0.17–1.06)		0.42 (0.16–1.12)
Employment					
No	3	75	1		1
Yes	1	55	0.81 (0.28–2.28)		0.45 (0.14–1.42)
Psychiatric comorbidities					
No	6	78	1		1
Yes	14	257	0.71 (0.26–1.90)		1.07 (0.36–3.17)
Alcohol co-abuse					
No	13	222	1		1
Yes	7	113	1.06 (0.41–2.72)		0.94 (0.35–2.49)
Drugs co-abuse					
No	15	237	1		1
Yes	5	98	0.81 (0.28–2.28)		0.45 (0.14–1.42)
Opioid maintenance therapy					
No	12	236	1		1
Yes	8	99	1.59 (0.63–4.00)		1.53 (0.54–4.30)
PHB equivalents at admission (mg/day)					
> 300	5	87	1		1
≤ 300	15	248	1.05 (0.37–2.98)		0.91 (0.32–2.63)
Number of abused active substances					
** = **1	14	243	1		1
≥ 2	6	92	1.13 (0.42–3.03)		0.90 (0.29–2.83)
Plasma half-life					
Long	2	32	1		1
Intermediate	15	250	0.96 (0.21–4.39)		0.74 (0.12–4.38)
Short + very short	3	53	0.91 (0.14–5.71)		0.62 (0.08–4.90)
Formulation					
Tablets	5	118	1		1
Drops	12	174	1.73 (0.56–4.74)		1.89 (0.60–5.97)
Both	3	43	1.65 (0.38–7.18)		1.45 (0.26–8.19)
Administration route					
Intravenous	1	15	1		1
Oral	19	315	0.90 (0.11–7.22)		1.59 (0.18–13.76)
Both	0	5	–		–
Supportive BZD					
No	7	157	1		1
Yes	13	178	1.64 (0.64–4.21)		1.78 (0.67–4.76)
Supportive CDZ					
No	13	201	1		1
Yes	7	134	0.81 (0.31–2.08)		0.88 (0.33–2.33)
Supportive GBP					
No	9	200	1		1
Yes	11	135	1.81 (0.73–4.49)		1.49 (0.58–3.82)
Supportive TZD					
No	16	271	1		1
Yes	4	64	1.06 (0.34–3.27)		1.24 (0.39–3.99)
Supportive psychiatric therapy					
No	8	67	1		1
Yes	12	268	0.37 (0.15–0.95)		0.42 (0.15–1.12)

Adjustment by age, sex, phenobarbital equivalents, number of abused active substances, plasma half-life, and formulation

Opioid maintenance therapy: buprenorphine or methadone

*BZD* benzodiazepine, *CDZ* chlordiazepoxide, *CI* confidence interval, *GBP* gabapentin, *OR* odds ratio, *PHB* phenobarbital, *TZD* trazodone

 Hospitalisation length, PHB discharge dose, and BZD free status at discharge according to patients’ demographic and pharmacological characteristics are reported in Table 4. The probability of having a hospitalisation > 7 days was observed for patients who reported opioid maintenance therapy (OR 2.07, CI 1.20–3.58), and for those treated with more than 300 mg/day of PHB equivalents at hospital admission (OR 1.68, CI 1.03–2.72). A longer hospitalisation was also observed for patients concomitantly treated with PHB, BZD and GBP (OR 2.10, CI 1.02–4.33) during their hospital stay. The probability to be discharged with a PHB dose > 100 mg/day was lower for patients who abused BZD with a long plasmatic half-life (OR 0.17, CI 0.03–0.95) compared to those who abused BZD with short/very short BZD and/or ZD. Finally, the multivariate logistic regression showed a higher probability to be BZD free at discharge for patients who reported having an employment (OR 2.29, CI 1.00–5.24), for those who abused oral drops of BZD and ZD (OR 2.16, CI 1.30–3.59), and for those concomitantly treated with PHB, BZD and TZD (OR 2.86, CI 1.14–7.17) during their hospital stay. On the contrary, alcohol co-abuse was associated with a lower probability to be BZD free at discharge (OR 0.08, CI 0.05–0.15).

**Table 4:** Hospitalisation length, PHB discharge dose, and BZD free status at discharge by demographic and pharmacological characteristics

	Duration of hospitalisation (> 7 days)		PHB dose at discharge (> 100 mg)		BZD free status at discharge (Yes)	
	Univariate OR (95% CI)	Multivariate OR (95% CI)	Univariate OR (95% CI)	Multivariate OR (95% CI)	Univariate OR (95% CI)	Multivariate OR (95% CI)
Sex						
Men	1	1	1	1	1	1
Women	1.18 (0.77–1.81)	1.38 (0.86–2.20)	0.86 (0.39–1.93)	0.71 (0.29–1.72)	1.08 (0.69–1.68)	1.01 (0.58–1.75)
Age						
< 40 years	1	1	1	1	1	1
≥ 40 years	1.08 (0.70–1.66)	1.11 (0.69–1.77)	0.93 (0.41–2.11)	0.69 (0.27–1.76)	0.73 (0.47–1.15)	0.78 (0.44–1.37)
Employment						
No	1	1	1	1	1	1
Yes	0.98 (0.49–1.96)	1.02 (0.50–2.07)	1.21 (0.28–5.29)	1.43 (0.32–6.40)	1.32 (0.66–2.66)	2.29 (1.00–5.24)
Psychiatric comorbidity						
No	1	1	1	1	1	1
Yes	0.96 (0.59–1.59)	1.01 (0.59–1.74)	1.79 (0.77–4.19)	2.31 (0.90–5.92)	0.90 (0.53–1.50)	1.05 (0.56–1.99)
Alcohol co-abuse						
No	1	1	1	1	1	1
Yes	0.97 (0.62–1.51)	1.01 (0.64–1.60)	0.57 (0.25–1.27)	0.60 (0.26–1.38)	0.12 (0.07–0.19)	0.08 (0.05–0.15)
Drugs co-abuse						
No	1	1	1	1	1	1
Yes	1.08 (0.68–1.72)	0.89 (0.51–1.54)	0.75 (0.32–1.75)	0.51 (0.18–1.40)	1.02 (0.63–1.64)	0.76 (0.40–1.47)
Opioid maintenance therapy						
No	1	1	1	1	1	1
Yes	1.77 (1.10–2.85)	2.07 (1.20–3.58)	1.89 (0.69–5.14)	2.66 (0.87–8.12)	1.59 (0.97–2.61)	1.74 (0.92–3.32)
PHB equivalents at admission (mg/day)						
≤ 300	1	1	1	1	1	1
> 300	1.63 (1.01–2.63)	1.68 (1.03–2.72)	-	-	1.42 (0.87–2.32)	1.43 (0.87–2.34)
Number of abused active substances						
= 1	1	1	1	1	1	1
≥ 2	1.02 (0.63–1.63)	1.07 (0.63–1.81)	0.85 (0.36–2.02)	0.46 (0.14–1.47)	1.25 (0.76–2.05)	1.03 (0.59–1.78)
Plasmatic half-life						
Short + very short	1	1	1	1	1	1
Intermediate	0.82 (0.39–1.71)	0.69 (0.30–1.60)	1.87 (0.51–6.95)	0.66 (0.14–3.15)	1.46 (0.70–3.02)	1.42 (0.61–3.27)
Long	1.20 (0.50–2.92)	1.12 (0.42–2.99)	0.44 (0.11–1.75)	0.17 (0.03–0.95)	0.87 (0.37–2.06)	0.82 (0.31–2.17)
Formulation						
Tablets	1	1	1	1	1	1
Drops	0.95 (0.60–1.51)	0.88 (0.54–1.44)	1.05 (0.43–2.54)	1.56 (0.50–4.86)	1.90 (1.18–3.06)	2.16 (1.30–3.59)
Both	1.06 (0.53–2.13)	1.00 (0.44–2.26)	0.83 (0.24–2.83)	0.58 (0.12–2.92)	1.07 (0.54–2.14)	0.73 (0.31–1.71)
Administration route						
Intravenous	1	1	1	1	1	1
Oral	1.10 (0.40–3.04)	1.07 (0.37–3.07)	3.09 (0.33–28.7)	4.12 (0.16–106)	0.42 (0.12–1.49)	0.46 (0.12–1.71)
Both	3.11 (0.28–34.42)	3.75 (0.32–44.15)	–	–	0.15 (0.02–1.37)	0.14 (0.01–1.31)
Supportive psychiatric therapy						
No	1	1	1	1	1	1
Yes	1.63 (0.98–2.73)	1.53 (0.90–2.60)	2.11 (0.90–4.94)	2.04 (0.65–6.43)	1.39 (0.83–2.35)	1.35 (0.78–2.33)

Opioid maintenance therapy: buprenorphine or methadone

Adjustment by age, sex, phenobarbital equivalents, number of abused active substances, half-life, and formulation

*BZD* benzodiazepines, *CI* confidence interval, *GBP* gabapentin, *OR* odds ratio, *PHB* phenobarbital, *TZD* trazodone

Supplementary Tables 1 and 2 report characteristics (number of active principles, type, plasma half-life, formulation, and administration route) of abused BZD and ZD in our cohort, with a focus on their total daily intake

## Discussion

This retrospective observational study highlights new insights on PHB use as a detoxification treatment in BZD use disorder patients. To the best of our knowledge, this study is the largest population-based investigation on the use of PHB for BZD withdrawal. Furthermore, this is the first study which also includes several ZD use disorder patients.

Considering PHB tolerability, only one patient developed an ADR, manifesting a non-serious dermatological reaction, and none developed severe withdrawal symptoms (i.e., delirium, seizures) nor clinically relevant sedation associable with the detoxification treatment. Moreover, no patients presented PHB acute intoxication requiring transferral to intensive care unit. Overall, discharge against medical advice was another undesirable outcome, accounting for a relatively low percentage of patients (5.6%), which is significantly lower than that reported in a brief article published in 2012 (17.1%) [12].

Comparing our clinical evidence with that already published in literature, we found only few studies on the use of PHB for BZD withdrawal [1], and two case reports on its use in ZD abusers [13]. Moreover, most of the identified studies were represented by anecdotal description of the clinical management of BZD withdrawal cases. Ravi et al. reported successful treatment of five alprazolam-dependent patients [14]. Each patient was given different doses of PHB (range 180–480 mg/day), and the length of treatment ranged from 9 to 19 days. Most of the patients underwent an initial BZD tapering before starting PHB treatment. Sullivan et al., examined 19 patients in a small randomised, double-blind controlled trial between clonazepam and PHB for the purpose of sedative-hypnotic tapering [15]. Clinicians used different doses of PHB or clonazepam based on BZD abuse reported by each patient, finding a superiority of BZD over barbiturates for symptoms of withdrawal but not of recurrent or rebound anxiety. Based on this evidence, it’s difficult to compare our results, both in terms of patients’ characteristics, type, and length of detoxification treatment, with the aforementioned clinical data, which is represented by outdated and relatively small samples.

On the other hand, of interest for an in depth comparison with our results is the evidence reported by Kawasaki et al. [12], who reviewed the medical records of 310 patients (median age 36 years) treated with a 3-day fixed-dose PHB taper for BZD dependence over a 5-year period. In their series, 57.1% and 25.2% of patients were on buprenorphine and methadone maintenance therapy, respectively. Although this occurrence was also observed in our sample with smaller percentages, BZD and/or ZD dependence is a common problem among patients on opioid maintenance therapy [16]. Both in the above-mentioned study and our analysis a PHB treatment was not associated with a negative outcome in BZD and/or ZD use disorder patients who are also treated for opioid dependence. The use of PHB should be carefully evaluated in patients with opioid maintenance therapy to avoid opioid withdrawal symptoms, especially in the case of a PHB fixed-dose regimen [12]. In fact, it is well known that PHB induces the cytochrome P450 enzyme, increasing methadone metabolism [17], thus reducing the abuse potential for PHB in this group compared to BZD [16]. Using BZD together with PHB allows a lower PHB daily dosage, thus reducing cytochrome P450 induction and, subsequently, the risk of opioid withdrawal symptoms. In fact, no increased doses of methadone were required during PHB treatment in our sample. However, we found that patients on buprenorphine or methadone receiving PHB treatment had a significantly longer hospital stay than others. This could be explained by the more complex clinical assistance level generally required by patients with opioid maintenance therapy [18]. Most of the patients managed by Kawasaki et al. were relatively young and did not have a concurrent medical illness. The median age observed in our population was 42 years (range 36–49), and the presence and the effect of other co-abuses and psychiatric comorbidities were taken into consideration from both a clinical and statistical point of view. We strongly believe that during the treatment of such patients, healthcare professionals should always address all clinical aspects underlying BZD and/or ZD use disorder, tailoring the detoxification treatment to each patient in the context of precision medicine, also in the field of clinical toxicology [19]. A fixed-dose PHB protocol should be used with caution in elderly subjects or those who are medically ill [12]. Conversely, using a protocol which can also include BZD allows to treat patients with alcohol-related liver disease by lowering the PHB daily dose in combination with specific BZD. It is well known, for example, that none of BZD are good to use in liver disease, but lorazepam is a safer choice for patients with liver function impairment [20]. Taking in consideration all these aspects, combine the PHB with long half-life BZD may help clinical toxicologists to treat those patients characterized by a more complicated clinical and psychological profile (i.e., patients undergoing maintenance therapy with methadone or buprenorphine, patients affected by liver disfunction, or those affected by severe psychiatric comorbidity). In our sample we observed a total of 191 subjects who required a supportive therapy, representing the subgroup with the highest clinical complexity. Furthermore, serum BZD concentration monitoring could help to make detoxification more reliable and effective [21], especially in this subgroup. Moreover, it should be noted that the detoxification treatment was used safely in pregnant women, who achieved complete detoxification, with no malformation or other pregnancy/neonatal negative outcomes. Finally, due to the impossibility of hospitalisation for COVID-19-related emergency measures, in 2020, a total of 6 patients were able to complete PHB detoxification as outpatients through ambulatory visits, demonstrating that this treatment is a valuable approach also in the context of the recent restrictions due to SARS-CoV-2 pandemic. In light of this, despite its spare use in past years for safety issues, if well driven, PHB detoxification treatment appears a valuable detoxification option for BZD and ZD use disorder patients.

The present analysis has some limitations and strengths. First, a long-term follow-up was also not analysed. Thus, it is not possible to draw conclusions about the effectiveness of PHB treatment in preventing long-term relapse. In addition, we did not find any demographic, clinical and pharmacological factors associated with “treatment failure”, in both men and women. This may be probably due to the total number of use disorder patients who referred to a single Toxicology Unit. In light of this, a multicentre analysis would be desirable. However, under the care of toxicology and addiction experts, PHB could be considered a useful and tolerable detoxification treatment for different types of use disorder patients, such as those under opioid maintenance therapy, polyabusers, medical ill patients, and pregnant women.

## Conclusions

BZD and/or ZD abuse and dependence have become clinically relevant during the last decades. Not many progresses have been made to find effective, safe, and manageable treatments for BZD and/or ZD withdrawal management and detoxification. Our study suggests that PHB, despite not being a recent detoxification option, can be used safely in clinical practice. Further research should evaluate its effectiveness compared to other existing treatments both in achieving a rapid detoxification and maintaining long-term abstinence.

## Supplementary Information

Below is the link to the electronic supplementary material.

Supplementary file1 (DOCX 24 KB)

## References

[1] Fluyau D, Revadigar N, Manobianco BE (2018). Challenges of the pharmacological management of benzodiazepine withdrawal, dependence, and discontinuation. Ther Adv Psychopharmacol.

[2] Brett J, Murnion B (2015). Management of benzodiazepine misuse and dependence. Aust Prescr.

[3] Lombardi N, Bettiol A, Crescioli G (2020). Risk of hospitalisation associated with benzodiazepines and z-drugs in Italy: a nationwide multicentre study in emergency departments. Intern Emerg Med.

[4] Milani SA, Raji MA, Chen L, Kuo YF (2021). Trends in the use of benzodiazepines, Z-hypnotics, and serotonergic drugs among US women and men before and during the COVID-19 pandemic. JAMA Netw Open.

[5] Schmitz A (2016). Benzodiazepine use, misuse, and abuse: a review. Ment Heal Clin.

[6] (2014) Guidance for the use and reduction of misuse of benzodiazepines and other hypnotics and anxiolytics in general practice. Available at: https://www.addictionprofessionals.org.uk/guidance-for-the-use-and-reduction-of-misuse-of-benzodiazepines. Accessed 8 April 2022

[7] Alexander B, Perry PJ (1991). Detoxification from benzodiazepines: schedules and strategies. J Subst Abuse Treat.

[8] (2021) ATC/DDD Index 2021. Available at: https://www.whocc.no/atc_ddd_index/ . Accessed 8 April 2022

[9] (2007) Sedative-hypnotic equivalency chart. Available at: https://physicians.utah.edu/echo/pdfs/sedative-hypnotic-equivalency-chart.pdf. Accessed 8 April 2022

[10] (2007) Benzodiazepine equivalence table. Available at: https://www.benzo.org.uk/bzequiv.htm. Accessed 8 April 2022

[11] Italian Medicines Agency (2019) Summary of Product Characteristics (Gardenale^®^). Available at: https://farmaci.agenziafarmaco.gov.it/aifa/servlet/PdfDownloadServlet?pdfFileName=footer_008055_004556_RCP.pdf&retry=0&sys=m0b1l3. Accessed 8 April 2022

[12] Kawasaki SS, Jacapraro JS, Rastegar DA (2012). Safety and effectiveness of a fixed-dose phenobarbital protocol for inpatient benzodiazepine detoxification. J Subst Abuse Treat.

[13] Beyraghi N, Shamsi A, Farrokhian A (2016). Detoxification of high-dose zolpidem using phenobarbital and gabapentin: two case reports. Arch Psychiatry Psychother.

[14] Ravi NV, Maany I, Burke WM (1990). Detoxification with phenobarbital of alprazolam-dependent polysubstance abusers. J Subst Abuse Treat.

[15] Sullivan M, Toshima M, Lynn P, Roy-Byrne P (1993). Phenobarbital versus clonazepam for sedative-hypnotic taper in chronic pain patients. A pilot study. Ann Clin Psychiatry.

[16] Chen KW, Berger CC, Forde DP (2011). Benzodiazepine use and misuse among patients in a methadone program. BMC Psychiatry.

[17] Mccance-Katz EF, Sullivan LE, Nallani S (2009). Drug interactions of clinical importance among the opioids, methadone and buprenorphine, and other frequently prescribed medications: a review. Am J Addict.

[18] Meyer P, Loney GC, Ferguson SM (2020). One is not enough: understanding and modeling polysubstance use. Front Neurosci.

[19] Cook JC, Wu H, Aleo MD, Adkins K (2018). Principles of precision medicine and its application in toxicology. J Toxicol Sci.

[20] Peppers MP (1996). Benzodiazepines for alcohol withdrawal in the elderly and in patients with liver disease. Pharmacotherapy.

[21] Basińska-Szafrańska A (2021). Metabolic diversity as a reason for unsuccessful detoxification from benzodiazepines: the rationale for serum BZD concentration monitoring. Eur J Clin Pharmacol.

