# Supplementary material for: Phenobarbital use in benzodiazepine and z-drug detoxification: a single-centre 15-year observational retrospective study in clinical practice
Source: Intern Emerg Med. 2022 Apr 12;17(6):1631–40. doi: 10.1007/s11739-022-02976-0 (PMC9001824; doi:10.1007/s11739-022-02976-0)
Supplement: Supplementary file 1 — Supplementary file1 (DOCX 24 KB) [file 11739_2022_2976_MOESM1_ESM.docx]

**Supplementary Table 1** Benzodiazepine and z-drug characteristics.

|  | **Total**  **N = 355 (%)** | **Men**  **N = 203 (%)** | **Women**  **N = 152 (%)** | ***p-value*** |
| --- | --- | --- | --- | --- |
| Only one BZD (n=257) | | | | |
| Diazepam equivalents (mg/day), median (IQR) | 250 (100-500) | 250 (125-500) | 250 (100-500) | 0.169 |
| Months of abuse, median (IQR) | 24 (12-72) | 24 (10-60) | 24 (12-96) | 0.447 |
| Two BZD (n=52) | | | | |
| Diazepam equivalents (mg/day), median (IQR) | 300 (130-530) | 315 (156.25-779.5) | 300 (125-530) | 0.602 |
| Months of abuse, median (IQR) | 24 (12-48) | 24 (12-48) | 24 (12-48) | 0.882 |
| Three BZD (n=21) | | | | |
| Diazepam equivalents (mg/day), median (IQR) | 631.25 (500-800) | 622.5 (420-685) | 768 (500-970) | 0.482 |
| Months of abuse, median (IQR) | 36 (12-84) | 60 (24-84) | 12 (10-78) | 0.279 |
| Only one ZD (n=12) | | | | |
| Diazepam equivalents (mg/day), median (IQR) | 150 (50-300) | 300 (200-375) | 100 (45-150) | 0.102 |
| Months of abuse, median (IQR) | 12 (6-54) | 48 (7.5-89) | 10 (6-23) | 0.670 |
| BZD and ZD association (n=13) | | | | |
| Diazepam equivalents (mg/day), median (IQR) | 115 (108-172.5) | 140.25 (97.75-1101.25) | 115 (115-115) | 1.000 |
| Months of abuse, median (IQR) | 17 (7-48) | 9.5 (5.5-36) | 24 (17-48) | 0.140 |
| Type of active substance | | | | |
| Alprazolam | 59 (16.62) | 22 (10.84) | 37 (24.34) |  |
| Bromazepam | 23 (6.48) | 8 (3.94) | 15 (9.87) |  |
| Brotizolam | 6 (1.69) | 6 (2.96) | 0 (0) |  |
| Clonazepam | 42 (11.83) | 25 (12.31) | 17 (11.18) |  |
| Chlordiazepoxide | 8 (2.25) | 6 (2.96) | 2 (1.32) |  |
| Delorazepam | 16 (4.51) | 5 (2.46) | 11 (7.23) |  |
| Diazepam | 38 (10.70) | 24 (11.82) | 14 (9.21) |  |
| Etizolam | 1 (0.28) | 1 (0.49) | 0 (0) |  |
| Flurazepam | 3 (0.84) | 2 (0.98) | 1 (0.66) |  |
| Lorazepam | 89 (25.07) | 61 (30.05) | 28 (18.42) |  |
| Lormetazepam | 139 (39.15) | 84 (41.38) | 55 (36.18) |  |
| Midazolam | 2 (0.56) | 2 (0.98) | 0 (0) |  |
| Triazolam | 16 (4.51) | 11 (5.42) | 5 (3.29) |  |
| Zolpidem | 23 (6.48) | 10 (4.93) | 13 (8.55) |  |
| Zopiclone | 2 (0.56) | 2 (0.98) | 0 (0) |  |
| Plasma half-life | | | | |
| Long | 56 (15.77) | 33 (16.26) | 23 (15.13) | 0.296 |
| Intermediate | 265 (74.65) | 151 (74.38) | 114 (75) |  |
| Short | 4 (1.13) | 4 (1.97) | 0 (0) |  |
| Very short | 30 (8.45) | 15 (7.39) | 15 (9.87) |  |
| Formulation | | | | |
| Tablets | 123 (34.65) | 79 (38.92) | 44 (28.95) | 0.133 |
| Drops | 186 (52.39) | 98 (48.28) | 88 (57.89) |  |
| Both | 46 (12.96) | 26 (12.81) | 20 (13.16) |  |
| Administration route | | | | |
| Oral | 334 (94.08) | 185 (91.13) | 149 (98.03) | 0.023 |
| Intravenous | 16 (4.51) | 14 (6.90) | 2 (1.32) |  |
| Both | 5 (1.41) | 4 (1.97) | 1 (0.66) |  |

BZD= benzodiazepines; IQR= interquartile range; SD= standard deviation; ZD= z-drugs

**Supplementary Table 2** Total daily intake of benzodiazepine and z-drug.

| Active substance | Registered DDD  (mg/day) | Conversion factor to diazepam | Diazepam equivalents (mg/day)  Mean (±SD) | | PHB equivalents (mg/day)  Mean (±SD) | | *p-value* |
| --- | --- | --- | --- | --- | --- | --- | --- |
|  |  |  | **Men** | **Women** | **Men** | **Women** |  |
| Alprazolam | 1 | 20 | 125.75 ± 134.53 | 237.91 ± 362.40 | 377.25 ± 403.59 | 713.74 ± 1087.21 | 0.190 |
| Bromazepam | 10 | 2 | 53.86 ± 33.37 | 73.2±46.14 | 161.57 ± 100.11 | 219.6 ± 138.41 | 0.334 |
| Brotizolam | 0.25 | 30 | 34.5 ± 43.92 | - | 103.5 ± 131.77 | - | - |
| Clonazepam | 8 | 20 | 787 ± 753.52 | 459 ± 419.94 | 2361 ± 2260.55 | 1377 ± 1259.82 | 0.136 |
| Chlordiazepoxide | 30 | 0.25 | 19 ± 11.49 | 16 ± 5.66 | 57 ± 34.47 | 48 ± 16.97 | 0.754 |
| Delorazepam | 3 | 10 | 122.2 ± 73.61 | 137 ± 156.45 | 366.6 ± 220.85 | 411 ± 469.35 | 0.844 |
| Diazepam | 10 | 1 | 53.67 ± 50.46 | 67.75 ± 85.22 | 161.02 ± 151.38 | 203.25 ± 255.64 | 0.531 |
| Etizolam | - | 10 | 75 ± 0.0 | - | 225 ± 0.0 | - | - |
| Flurazepam | 30 | 0.5 | 15 ± 0.0 | 15 ± 0.0 | 45 ± 0.0 | 45 ± 0.0 | - |
| Lorazepam | 2.5 | 10 | 263.53 ± 189.29 | 182.1 ± 200.90 | 790.60 ± 567.88 | 546.3 ± 602.70 | 0.081 |
| Lormetazepam | 1 | 10 | 586.57 ± 482.48 | 538.42 ± 366.37 | 1759.72 ± 1447.45 | 1615.28 ± 1099.12 | 0.534 |
| Midazolam | 15 | 2 | 40 ± 0.0 | - | 120 ± 0.0 | - | - |
| Triazolam | 0.25 | 40 | 161.82 ± 138.41 | 66.00 ± 43.93 | 485.45 ± 415.22 | 198.00 ± 131.79 | 0.159 |
| Zolpidem | 10 | 0.5 | 34.00 ± 41.44 | 61.25 ± 68.95 | 102.00 ± 124.33 | 183.75 ± 206.86 | 0.460 |
| Zopiclone | 7.5 | 0.5 | 175.89 ± 177.44 | 122.86 ± 150.74 | 527.68 ± 532.31 | 368.57 ± 452.23 | 0.558 |

IQR= interquartile range; PHB= phenobarbital; SD= standard deviation.
